# Current status of function‐preserving gastrectomy for gastric cancer

**DOI:** 10.1002/ags3.12430

**Published:** 2021-01-27

**Authors:** Toshiyuki Kosuga, Masahiro Tsujiura, Susumu Nakashima, Mamoru Masuyama, Eigo Otsuji

**Affiliations:** ^1^ Department of Surgery Saiseikai Shiga Hospital Ritto Japan; ^2^ Division of Digestive Surgery Department of Surgery Kyoto Prefectural University of Medicine Kyoto Japan

**Keywords:** function‐preserving gastrectomy, gastric cancer, proximal gastrectomy, pylorus‐preserving gastrectomy, subtotal gastrectomy

## Abstract

Early gastric cancer (EGC) has excellent postoperative survival outcomes; thus, one of the recent keywords in the treatment of EGC is “function‐preserving gastrectomy (FPG).” FPG reduces the extent of lymphadenectomy and gastric resection without compromising the long‐term prognosis. Proximal gastrectomy (PG) is an alternative to total gastrectomy (TG) for EGC in the upper‐third of the stomach, in which the gastric reservoir, gastric acid secretion, and intrinsic factors are maintained. Distal gastrectomy (DG) with a small remnant stomach, namely subtotal gastrectomy (STG), is another option for upper EGC, where the function of the cardia and fundus is preserved. Pylorus‐preserving gastrectomy (PPG) is a good alternative to DG for EGC in the middle‐third of the stomach, where pyloric function is preserved. Following elucidation of the markedly low incidences of possible metastasis to lymph node stations where dissection is omitted, the oncological safety of these FPG procedures was clarified. Nutritional advantages of PG or STG over TG have been reported; however, the standardized reconstruction methods after PG are yet to be established, and it is important to devise methods to prevent postoperative gastroesophageal reflux and anastomotic complications regardless of the reconstruction method. Nutritional benefits of PPG compared with DG have also been clarified, in which reducing postoperative gastric stasis is important. For the further spread of these FPG procedures, several issues, such as precise evaluation of preserved function, confirmation of oncological safety, and standardization of the technique, should be addressed in future prospective randomized controlled trials.

## Graphical abstract

Function‐preserving gastrectomy, such as proximal gastrectomy, subtotal gastrectomy, or pylorus‐preserving gastrectomy, for early gastric cancer is an attractive surgical procedure to maintain gastric function and postoperative quality of life. For these procedures to gain popularity, several issues, such as precise evaluation of preserved function, confirmation of oncological safety, and standardization of the technique, should be addressed in future prospective randomized controlled trials.

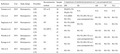

## 1 INTRODUCTION

Early gastric cancer (EGC) has a low incidence of lymph node metastasis and excellent postoperative survival outcomes; thus, recent keywords in the treatment of EGC are “minimally invasive gastrectomy (MIG)” and “function‐preserving gastrectomy (FPG).”^1^ FPG reduces the extent of lymphadenectomy and gastric resection without compromising the long‐term prognosis; thus, FPG can theoretically maintain the gastric function and postoperative quality of life (QOL) of patients.^1^ Proximal gastrectomy (PG) and distal gastrectomy (DG) with a small remnant proximal stomach, namely subtotal gastrectomy (STG), are alternatives to total gastrectomy (TG) for EGC in the upper‐third of the stomach, whereas pylorus‐preserving gastrectomy (PPG) is a good alternative to DG for EGC in the middle‐third of the stomach. Recently, these FPG procedures have been performed using an MIG such as laparoscopic or robotic gastrectomy.

Recent studies demonstrated the nutritional advantages of PG or STG over TG. The nutritional benefits of PPG compared with DG were also clarified. In this review article, we summarize the current status of FPG procedures, with a special focus on postoperative functional and nutritional outcomes.

## 2 Proximal gastrectomy (**PG)**

Due to the increasing incidence of proximal gastric cancer (GC), the demand for PG is also increasing.^2^ In PG, the gastric reservoir, gastric‐acid secretion, and intrinsic factors are maintained. On the other hand, patients undergoing PG may develop heartburn due to gastroesophageal reflux, which may lead to a poor postoperative QOL. Although there is no consensus on the optimal procedure, the choice of reconstruction method must be made in consideration of the prevention of gastroesophageal reflux and the guarantee of nutritional benefits.

### 2.1 Indications and oncological safety of PG

In patients with upper‐third EGC, metastasis to lymph node stations #4d/#5/#6 are rare; therefore, dissection of these nodes is considered unnecessary. Many studies reported that the long‐term oncological outcomes of PG were similar to those of TG.^3^, ^4^ Ichikawa et al^3^ reported that the overall survival (OS) rate of the PG group was similar to that of the TG group (5‐year survival rate, 95% vs 97%, respectively; *P* = .86). Accordingly, in the Japanese Gastric Cancer Treatment Guidelines (JGCTG),^5^ PG is recommended as an option for cT1N0 tumors in the upper‐third of the stomach, in which the size of the remnant stomach can be more than half of the original.

Considering the markedly low metastatic rates and therapeutic index at lymph node stations #4/#5/#6, even proximal advanced GC or esophagogastric junctional (EGJ) cancer <4 cm in diameter may be indicated for PG.^6^, ^7^ However, PG for such lesions is technically difficult because of the complete dissection of #11d (and #10) and anastomotic procedure in the mediastinum. Moreover, it is unclear whether wider excision on both the esophageal and gastric sides guarantees the nutritional benefits of PG. At this time, indications of PG for these lesions should be carefully considered.

### 2.2 Surgical procedures of PG

In PG, D1 lymphadenectomy includes lymph node stations #1/#2/#3a/#4sa/#4sb/#7, and stations #8a/#9/#11p are additionally included for D1+ lymphadenectomy.^5^ The right gastric and gastroepiploic vessels will be preserved. The hepatic and pyloric branches of the vagus nerve are routinely preserved, whereas its celiac branch is usually not preserved. The recent prospective phase II study (JCOG1401) confirmed the safety of laparoscopic PG (LPG).^8^ Considering the non‐inferiority of laparoscopic DG (LDG) to open DG in clinical stage I GC relapse‐free survival (RFS) confirmed by a phase III randomized controlled trial (RCT) (JCOG0912),^9^ LPG is now considered to be one of the standard treatments for cStage I GC.

There are two major reconstruction methods after PG: one is esophagogastrostomy (EG) and the other uses the small intestine. These procedures have their pros and cons, and the optimal method remains controversial^2^; therefore, at present, the method of reconstruction after PG is selected depending on the proficiency level of the surgeon at each facility. EG is a simple and physiological reconstruction method as it includes only one anastomotic site; however, a higher frequency of reflux esophagitis may develop postoperatively if additional anti‐reflux procedures are not performed together. Reconstruction methods using the small intestine include jejunal interposition (JI), double tract reconstruction (DT) and jejunal pouch interposition (JPI). In a Japanese questionnaire survey in 2010 regarding reconstruction methods after PG, the most common method was EG (48%), followed by JI (28%), DT (13%), and JPI (7%).^10^ However, regarding reconstructions using the small intestine, DT has recently gained popularity due to its easier laparoscopic approach. In fact, in JCOG1401, DT was performed in 45 (91.8%) patients and JI in only four patients (8.2%).^8^

### 2.3 Functional and nutritional outcomes of PG

An et al^11^ reported in 2008 that PG provides no nutritional advantages over TG because of a markedly higher rate of complications such as anastomotic stenosis and reflux esophagitis. However, in recent years, novel laparoscopic EG techniques, such as EG with fundoplication,^12^, ^13^, ^14^, ^15^ gastric tube reconstruction,^16^, ^17^ double flap technique (DFT),^18^, ^19^ and side overlap with fundoplication by Yamashita (SOFY)^20^ were reported with the reduced incidence of reflux esophagitis. Reconstruction methods using the small intestine have also been performed laparoscopically and have advantages for the prevention of reflux esophagitis.^21^, ^22^, ^23^ Although JPI is effective for preserving gastric function,^24^ it is complex, and may be associated with dilatation and stasis of the jejunal pouch.^25^ Table 1 shows the incidence of postoperative anastomotic stricture and reflux esophagitis in each reconstruction method after LPG.^12^, ^13^, ^14^, ^15^, ^16^, ^17^, ^18^, ^19^, ^20^, ^21^, ^22^, ^23^

**TABLE 1:** Recent studies examining the incidence of postoperative anastomotic stricture and reflux esophagitis in each reconstruction method after LPG

References	Year	Study design	Sample size (n)	Anastomotic stricture (%)	Reflux esophagitis (%)	
					Symptom	Endoscopy^a^
EG with fundoplication						
Komatsu et al	2020	Retrospective	23	4.3	0.0	0.0
Nishigori et al	2017	Retrospective	20	25.0	18.0	5.0
Kosuga et al	2015	Retrospective	25	16.0	12.0	9.1
Ahn et al	2013	Retrospective	50	12.0	32.0	N/A
EG (gastric tube reconstruction)						
Yasuda et al	2015	Retrospective	25	21.7	4.3	13.6
Mochiki et al	2014	Retrospective	41	14.6	N/A	9.8
EG (DFT)						
Hosoda et al	2019	Retrospective	40	17.5	17.5	8.3
Shoji et al	2019	Retrospective	147	8.3	N/A	4.2
EG (SOFY)						
Yamashita et al	2017	Retrospective	14	0.0	7.1	7.1
JI						
Kinoshita et al	2013	Retrospective	22	9.1	0.0	0.0
DT						
Jung et al	2017	Retrospective	92	3.3	1.1	N/A
Ahn et al	2014	Retrospective	43	4.7	4.7	0.0

Abbreviations: DFT, double flap technique; DT, double tract; EG, esophagogastrostomy; JI, jejunal interposition; LPG, laparoscopic proximal gastrectomy; N/A, not available; SOFY, Side overlap with fundoplication by Yamashita.

^a^ Grade ≥ B in Los Angeles classification.

Following the recent improvements in postoperative short‐term outcomes of laparoscopic procedures, nutritional benefits of LPG have been increasingly reported. Seven studies that assessed the nutritional advantages of LPG over laparoscopic TG (LTG) in terms of body weight (BW), hemoglobin (Hb), albumin (Alb), total protein (TP) and total lymphocyte count are presented in Table 2.^12^, ^14^, ^26^, ^27^, ^28^, ^29^, ^30^ Patients undergoing LPG had a significantly higher BW and Hb level than those undergoing LTG. Several studies reported the higher serum levels of iron and vitamin B12 after LPG.^22^, ^26^, ^31^ Not only maintenance of gastric acid but also food passage through the duodenum are important factors for nutrient absorption, especially iron. Based on the QOL analysis according to the post‐gastrectomy syndrome assessment scale‐45 (PGSAS‐45), Takiguchi et al^32^ reported that PG was better than TG in terms of BW loss, necessity of additional meals, diarrhea, and dumping symptoms. In addition, Inada et al^33^ reported that the diarrhea symptom subscale and necessity for additional meals scores were lower in patients with a remnant stomach of more than three‐quarters than in those with a remnant stomach two‐thirds the preoperative size; therefore, especially in EG, a large remnant stomach should be preserved to provide a better postoperative QOL.

**TABLE 2:** Studies examining the nutritional advantages of LPG over LTG

References	Year	Study design	Procedure	Reconstruction method	Sample size (n)	Comparisons of nutritional parameters (at 1 y postoperatively)				
						BW	Hb	Alb	TP	TLC
Nomura et al	2019	Retrospective	LPG	DT or JI	30	PG(DT) > TG PG(JI) > TG	N/A	N/A	N/A	N/A
			LTG		30					
Cho et al	2019	Retrospective	LPG	DT	38	N/A (BMI: PG = TG)	PG > TG (PG = TG at 2 y postoperatively)	PG = TG	PG = TG	PG = TG
			LTG		42					
Sugiyama et al	2018	Retrospective	LPG	DT	10	PG > TG (SMI: PG > TG)	PG = TG	PG = TG	N/A	PG = TG
			LTG		20					
Hayami et al	2017	Retrospective	LPG	EG (DFT)	43	PG > TG	PG > TG	PG = TG	PG > TG	N/A
			LTG		47					
Hosoda at al	2016	Retrospective (PSM)	LPG	EG	16	PG = TG	PG = TG (PG > TG at 2 y postoperatively)	PG = TG	PG = TG	PG = TG
			LTG		16					
Kosuga et al	2015	Retrospective	LPG	EG	25	PG > TG	PG = TG (PG > TG at 2 y postoperatively)	PG > TG	PG = TG	PG > TG
			LTG		52					
Ahn et al	2013	Retrospective	LPG	EG	50	PG = TG	PG = TG	PG = TG	PG = TG	PG = TG
			LTG		81					

Abbreviations: Alb, albumin; BMI, body mass index; BW, body weight; DFT, double flap technique; DT, double tract; EG, esophagogastrostomy; Hb, hemoglobin; JI, jejunal interposition; LPG, laparoscopic proximal gastrectomy; LTG, laparoscopic total gastrectomy; N/A, not available; PG, proximal gastrectomy; PSM, propensity score matching; SMI, skeletal muscle index; TG, total gastrectomy; TLC, total lymphocyte count; TP, total protein.

## 3 Subtotal gastrectomy (STG)

In some patients with EGC in the upper gastric body, there is distance from the tumor to the EGJ. In selected patients, DG with small remnant proximal stomach, namely STG, can be applied, in which the function of the cardia is preserved. STG is usually performed via the laparoscopic approach (LSTG).

### 3.1 Indications and oncological safety of STG

The basic indications for LSTG are as follows^1^: (i) EGC diagnosed as cT1N0M0; (ii) tumor located in or involving the upper‐third of the stomach; (iii) remaining distance from the tumor to EGJ of less than 5 cm,; and (iv) remnant gastric stump 2‐3 cm away from EGJ. When an oncologically safe distance from the tumor to EGJ cannot be secured, LPG or LTG is an alternative procedure. Preserving a proximal stomach may raise two oncological concerns: one is the positive margin and the other is possible lymph node metastasis to stations #2/#4sa. Kano et al^34^ reported that LSTG may be an oncologically acceptable procedure for cT1N0 GC in the upper gastric body because no patients undergoing LSTG, LPG, or LTG had metastasis at stations #2/#4sa or developed GC recurrence. Patients with LSTG had 3‐year OS and RFS rates similar to those with LPG or LTG.^34^ However, the width of the pathological margin in LSTG was significantly shorter than that in LPG or LTG^34^; thus, in LSTG, more meticulous preoperative and intraoperative diagnosis is essential to secure the negative margin. It is a very difficult question whether to apply either LSTG or LPG to EGC confined to the upper gastric body. If both procedures can be performed with technical and oncological safety, LPG may be advantageous in terms of wider surgical margin and larger size of the remnant stomach.

### 3.2 Surgical procedures of STG

Based on the dissection range in DG, D1 lymphadenectomy includes lymph node stations #1/#2/#3/#4sb/#4d/#5/#6/#7, and stations #8a/#9 are additionally included for D1+ lymphadenectomy in STG.^5^ Station #11p is often dissected because it is in close proximity to the primary tumor. Under intraoperative endoscopic observation, the stomach is transected using an endoscopic linear stapler on the oral side of the preoperative markings, leaving a small remnant proximal stomach. Intraoperative frozen‐section analysis is performed to confirm the cancer‐negative gastric stump. For achieving a technically and oncologically safe gastric transection in LSTG, Kawakatsu et al found the usefulness of preoperative determination of the tumor site with clips in combination with intraoperative endoscopy, and Kamiya et al introduced a new marking technique called endoscopic cautery marking.^35^, ^36^ Roux‐en‐Y (RY) reconstruction is created via an antecolic route. For gastrojejunostomy, a 25‐mm circular stapler (Orvil Covidien, Mansfield, MA, USA) is initially used to not interrupt the blood supply from the short gastric arteries by stapling^1^; however, an endoscopic linear stapler has also been used recently without negatively affecting the postoperative short‐term outcomes.^37^

### 3.3 Functional and nutritional outcomes of STG

Three studies examining the nutritional advantages of LSTG over LTG or LPG in terms of BW, Hb, Alb, TP, and prognostic nutritional index (PNI) are presented in Table 3.^37^, ^38^, ^39^ Kosuga et al^39^ first reported that patients undergoing LSTG had a significantly higher postoperative BW, and serum Alb and TP levels, than those undergoing LTG. Kano et al^37^ reported that the postoperative BW, and serum Alb and TP levels, were comparable between the two procedures, but postoperative Hb concentrations were higher after LPG‐DFT than after LSTG. Considering the difference in food passage routes between the two procedures, their results are reasonable. Furukawa et al^38^ demonstrated that patients with LSTG had significantly higher postoperative BW and Hb concentrations than those with LTG, and LSTG resulted in better serum Alb and PNI levels than LPG. Although the reason why LSTG was nutritionally better than LPG remains unclear, the improved nutritional status after LSTG, despite a small remnant stomach, may result from preservation of the gastric fundus, the primary location of ghrelin secretion.^40^ Yasufuku et al^41^ reported that a small remnant stomach after LSTG was worth preserving considering the postoperative nutritional maintenance even though the postoperative BW and Hb levels were slightly lower than those in patients with a standard‐size remnant stomach after conventional LDG.

**TABLE 3:** Studies examining the nutritional advantages of LSTG over LTG or LPG

References	Year	Study design	Procedure	Sample size (n)	Comparisons of nutritional parameters (at 1 y postoperatively)				
					BW	Hb	Alb	TP	PNI
Kano et al	2020	Retrospective	LSTG	110	STG = PG	STG = PG (STG < PG at 2 and 3 y postoperatively)	STG = PG	STG = PG	N/A
			LPG (EG‐DFT)	51					
Furukawa et al	2018	Retrospective	LSTG	38	STG > TG	STG > TG	N/A	N/A	STG = TG
			LTG	48					
			LPG (EG or DT)	27	STG = PG	N/A	STG > PG	N/A	STG > PG
Kosuga et al	2014	Retrospective	LSTG	57	STG > TG	N/A	STG > TG	STG > TG	N/A
			LTG	110					

Abbreviations: Alb, albumin; BW, body weight; DFT, double flap technique; DT, double tract; EG, esophagogastrostomy; Hb, hemoglobin; LPG, laparoscopic proximal gastrectomy; LSTG, laparoscopic subtotal gastrectomy; LTG, laparoscopic total gastrectomy; N/A, not available; PG, proximal gastrectomy; PNI, prognostic nutritional index; STG, subtotal gastrectomy; TG, total gastrectomy; TP, total protein.

## 4 Pylorus‐preserving gastrectomy (PPG)

PPG was introduced as a surgical procedure for EGC in the middle‐third of the stomach designed to preserve pyloric function and maintain a better postoperative QOL.^42^ PPG has several functional and nutritional benefits, with a lower incidence of post‐gastrectomy syndromes, such as dumping syndrome and bile reflux, compared with conventional DG with Billroth I (BI) reconstruction.

### 4.1 Indications and oncological safety of PPG

In cT1N0 tumors in the middle‐third of the stomach, metastasis to lymph node stations #5/#6i is rare; therefore, dissection of these nodes can be omitted. Previous studies reported low incidences of supra‐ and infra‐pyloric lymphatic metastasis (#5 and #6), ranging from 0.00% to 0.45% and from 0.45% to 2.60%, respectively, for EGC in the middle‐third of the stomach.^43^, ^44^, ^45^ Mizuno et al^44^ reported that none of 117 patients with EGC in the middle‐third of the stomach had metastasis to lymph node station #6i, suggesting that lymphadenectomy along the infra‐pyloric artery is dispensable in PPG. Indeed, several reports described satisfactory 5‐year OS rates of PPG (96.3%‐98.4%), comparable to those after conventional DG.^46^, ^47^, ^48^ Accordingly, in the JGCTG, PPG is recommended as an option for cT1N0 tumors in the middle portion of the stomach with a distal tumor border of at least 4 cm proximal to the pylorus.^5^

### 4.2 Surgical procedures of PPG

In PPG, D1 lymphadenectomy includes lymph node stations #1/#3/#4sb/#4d/#6/#7, and stations #8a/#9 are additionally included for D1+ lymphadenectomy.^5^ As the roots of the right gastric artery and vein are routinely left intact, these vessels are transected after the first branch. The infra‐pyloric artery and vein should be preserved. The right gastroepiploic artery and vein are transected after bifurcation of the infra‐pyloric vessels. The hepatic and pyloric branches of the vagus nerve are routinely preserved, and its celiac branch is preserved in some cases. Initially, the distal transection line was made 1.5 cm proximal to the pyloric ring; however, meal stasis was common. The kinetics of gastric emptying were investigated and the length of the pyloric cuff was gradually increased.^49^, ^50^ Thus, a 3‐ to 4‐cm pyloric cuff is generally preserved in PPG. PPG is now usually performed via the laparoscopic approach (LPPG).^48^, ^51^ In laparoscopy‐assisted surgery, gastro‐gastro anastomosis is extracorporeally performed directly from the small upper abdominal middle incision employing hand‐sewn techniques.^51^ Recently, novel techniques for intracorporeal anastomosis such as the delta‐shaped method and the piercing method were introduced with excellent postoperative outcomes.^52^, ^53^.

### 4.3 Functional and nutritional outcomes after PPG

Three studies after 2014 that assessed the functional and nutritional advantages of PPG over DG are presented in Table 4.^45^, ^54^, ^55^ Suh et al^45^ reported that decreases in serum TP and Alb levels 1 to 6 months postoperatively were significantly smaller in LPPG than in LDG, although delayed gastric emptying was more frequent in LPPG than in LDG (7.8% vs 1.7%). The 3‐year cumulative incidence of gallstones was significantly less in LPPG than in LDG (0% vs 6.5%).^45^ Fujita et al^54^ reported BW loss of −6.9% in the PPG group and −7.9% in the DG with BI group (*P* = .052). Regarding QOL analysis according to the PGSAS‐45, two multicenter analyses revealed significantly better outcomes regarding dumping syndrome and diarrhea after PPG than after DG.^54^, ^55^ Namikawa et al^56^ reported that the size of the proximal gastric remnant significantly affects the change in BW, scores for dissatisfaction at meals, and dissatisfaction for daily life subscale; thus, preservation of a sufficient proximal gastric remnant is recommended in PPG. Wang et al^57^ reported that preservation of the hepatic branch of the vagus nerve reduced the risk of gallstone formation after LPPG, whereas Fujita et al^54^ reported that preservation of the celiac branch of the vagus nerve was an independent factor predicting diarrhea and dumping. Meanwhile, Furukawa et al^58^ found no definite functional impact of preservation of the celiac branch of the vagus nerve; therefore, whether or not to preserve the celiac branch is now under debate. PPG is sometimes associated with postoperative gastric stasis. Kiyokawa et al^59^ reported that preservation of the infra‐pyloric vein helped in preventing postoperative gastric stasis after LPPG by reducing venous stasis and edema of the pyloric cuff; thus, it should be preserved in addition to the infra‐pyloric artery. Takahashi et al^60^ recently reported that age ≥61 years, diabetes mellitus, and postoperative intraabdominal infection are significantly related to postoperative gastric stasis, and those who develop gastric stasis have poorer nutritional and functional outcomes even at 1 year postoperatively. Therefore, more strict indications and safer surgery are required to prevent postoperative gastric stasis. Regarding the indications, Tsujiura et al^61^ noted that even overweight/obese patients were good candidates for PPG based on postoperative nutritional maintenance. An ongoing Korean multicenter randomized controlled trial (KLASS‐04) comparing LPPG and LDG may provide more clear evidence about the advantages and oncological safety of PPG.

**TABLE 4:** Recent important studies examining the functional and nutritional benefits of LPPG over LDG

References	Year	Study design	Procedure	Sample size (n)	Symptom (PGSAS subscale)			Gastric stasis	Gallstone formation	Nutritional parameters		
					Esophageal reflux	Dumping	Diarrhea			BW change	TP	Alb
Hosoda et al	2017	Retrospective (PSM)	LPPG	32	2.0	1.5	1.9			93.1%		
			LDG‐BI	32	1.8	2.0	2.4			91.8%		
			*P* value		.570	.042	.028			.450		
Fujita et al	2016	Retrospective	LPPG	313	1.7	1.8	1.8			93.1%		
			LDG‐BI	909	1.7	2.0	2.1			92.1%		
			*P* value		≥.100	.003	<.001			.052		
Suh et al	2014	Retrospective	LPPG	116				7.8%	0%		+3.9%	+4.0%
			LDG	176				1.7%	6.5%		−0.2%	−0.6%
			*P* value					.015	.038		.008^a^	.012^a^

Abbreviations: Alb, albumin; BI, Billroth I; BW, body weight; LDG, laparoscopic distal gastrectomy; LPPG, laparoscopic pylorus‐preserving gastrectomy; PGSAS, post‐gastrectomy syndrome assessment scale; PSM, propensity score matching; TP, total protein.

^a^ Calculated by analysis of covariance (ANCOVA) adjusted by preoperative parameters.

## 5 CONCLUSIONS AND FUTURE PERSPECTIVES

FPG procedures, such as PG, STG, and PPG, for EGC are attractive surgical procedures to maintain the gastric function and postoperative QOL of patients; however, there is little evidence from prospective trials supporting their usefulness compared with other surgical procedures. The lack of consensus on the optimal reconstruction method after PG is a major problem. Therefore, several issues, such as precise evaluation of preserved function, confirmation of oncological safety, and standardization of the technique, need to be strictly addressed in prospective well‐designed RCTs. In recent years, evidence supporting the clinical safety and efficacy of sentinel node navigation surgery for EGC has accumulated.^62^ In the near future, segmental gastrectomy, local resection, and endoscopic submucosal dissection with sentinel basin dissection may become the standard FPG procedures for EGC.

## DISCLOSURE

The authors declare no conflict of interests for this article.
